# Clinical Management in Treatment-Resistant Schizophrenia: A Case of Clozapine-Associated Myocarditis and Transition to Alternative Antipsychotic Therapy

**DOI:** 10.1192/j.eurpsy.2026.12188

**Published:** 2026-07-31

**Authors:** K. Çakmak, Y. Kavla, Ö. F. Demirel

**Affiliations:** Psychiatry Department, Istanbul University- Cerrahpaşa, Faculty of Medicine, İstanbul, Türkiye

## Abstract

**Introduction:**

Schizophrenia is a chronic psychiatric disorder with disturbances in thought, emotion, and behavior. Antipsychotics are first-line treatments, yet ~30% of patients show treatment-resistant schizophrenia (TRS), requiring clozapine. Clozapine offers substantial benefits but carries risks including agranulocytosis, myocarditis, and sedation, sometimes necessitating discontinuation. This case describes a TRS patient who developed suspected clozapine-associated myocarditis. Such complications may not only restrict its utilization but may also necessitate a therapeutic switch. Written informed consent was obtained from the patient.

**Objectives:**

To highlight clozapine limitations, emphasize adverse effect monitoring, and underscore the need for alternative strategies when clozapine is contraindicated.

**Methods:**

Clinical evaluation was conducted using standardized instruments, including the Positive and Negative Syndrome Scale (PANSS). Magnetic resonance imaging (MRI) was reviewed. Alternative therapeutic strategies were planned, taking into account both efficacy and tolerability.

**Results:**

The patient, a 47-year-old divorced female with two children under paternal custody and legally under her father’s guardianship, was admitted due to absconding from home, disruptive behaviors and lack of insight. Mental status revealed preserved self-care, irritability, and persecutory, erotomanic, and referential delusions. Her first psychiatric consultation was 16 years earlier for depressive symptoms, initially diagnosed as bipolar disorder with limited response. Prior regimens included olanzapine, risperidone, quetiapine, venlafaxine, and duloxetine.

On admission, amisulpride was titrated to 1000 mg/day with insufficient response. Clozapine 50 mg/day was added, titrated to 200 mg/day, alongside planned ECT. Sedation and tachycardia emerged, requiring metoprolol. On day 16, she developed chest pain, palpitations, and fever (38.1°C). ECG showed sinus tachycardia; troponin was elevated. Coronary CTA excluded coronary artery disease. Clozapine was discontinued, ECT paused, and treatment was transitioned to risperidone. She received 10 ECT sessions. After 49 days, she was discharged on risperidone 8 mg/day, quetiapine XR 200 mg/day, and aripiprazole 400 mg injection. PANSS improved from 78 to 69, with partial remission.

**Conclusions:**

In many patients, treatment resistance requires alternatives such as clozapine and ECT, yet adverse effects may limit clozapine use. This report highlights the need for vigilant monitoring and management of clozapine in TRS and consideration of alternative strategies when contraindicated.

**Disclosure of Interest:**

None Declared

